# Association Study of Common Genetic Variants and HIV-1 Acquisition in 6,300 Infected Cases and 7,200 Controls

**DOI:** 10.1371/journal.ppat.1003515

**Published:** 2013-07-25

**Authors:** Paul J. McLaren, Cédric Coulonges, Stephan Ripke, Leonard van den Berg, Susan Buchbinder, Mary Carrington, Andrea Cossarizza, Judith Dalmau, Steven G. Deeks, Olivier Delaneau, Andrea De Luca, James J. Goedert, David Haas, Joshua T. Herbeck, Sekar Kathiresan, Gregory D. Kirk, Olivier Lambotte, Ma Luo, Simon Mallal, Daniëlle van Manen, Javier Martinez-Picado, Laurence Meyer, José M. Miro, James I. Mullins, Niels Obel, Stephen J. O'Brien, Florencia Pereyra, Francis A. Plummer, Guido Poli, Ying Qi, Pierre Rucart, Manj S. Sandhu, Patrick R. Shea, Hanneke Schuitemaker, Ioannis Theodorou, Fredrik Vannberg, Jan Veldink, Bruce D. Walker, Amy Weintrob, Cheryl A. Winkler, Steven Wolinsky, Amalio Telenti, David B. Goldstein, Paul I. W. de Bakker, Jean-François Zagury, Jacques Fellay

**Affiliations:** 1 School of Life Sciences, École Polytechnique Fédérale de Lausanne, Lausanne, Switzerland; 2 Institute of Microbiology, University Hospital Center and University of Lausanne, Lausanne, Switzerland; 3 Program in Medical and Population Genetics, Broad Institute of MIT and Harvard, Cambridge, Massachusetts, United States of America; 4 Laboratoire Génomique, Bioinformatique, et Applications, EA4627, Chaire de Bioinformatique, Conservatoire National des Arts et Métiers, Paris, France; 5 ANRS Genomic Group (French Agency for Research on AIDS and Hepatitis), Paris, France; 6 Center for Human Genetic Research, Massachusetts General Hospital, Harvard Medical School, Boston, Massachusetts, United States of America; 7 Department of Neurology, Rudolf Magnus Institute of Neuroscience, University Medical Center Utrecht, Utrecht, The Netherlands; 8 Bridge HIV, San Francisco Department of Public Health, San Francisco, California, United States of America; 9 Cancer and Inflammation Program, Laboratory of Experimental Immunology, SAIC Frederick, Inc., Frederick National Laboratory for Cancer Research, Frederick, Maryland, United States of America; 10 Ragon Institute of MGH, MIT and Harvard, Boston, Massachusetts, United States of America; 11 Department of Surgery, Medicine, Dentistry and Morphological Sciences University of Modena and Reggio Emilia School of Medicine, Modena, Italy; 12 AIDS Research Institute IrsiCaixa, Institut d'Investigació en Ciències de la Salut Germans Trias i Pujol, Universitat Autònoma de Barcelona, Badalona, Spain; 13 Department of Medicine, University of California, San Francisco, California, United States of America; 14 Department of Statistics, University of Oxford, Oxford, United Kingdom; 15 University Division of Infectious Diseases, Siena University Hospital, Siena, Italy; 16 Institute of Clinical infectious Diseases, Università Cattolica del Sacro Cuore, Roma, Italy; 17 Infections and Immunoepidemiology Branch, Division of Cancer Epidemiology and Genetics, National Cancer Institute, Rockville, Maryland, United States of America; 18 Vanderbilt University School of Medicine, Nashville, Tennessee, United States of America; 19 Department of Microbiology, University of Washington, Seattle, Washington, United States of America; 20 Cardiovascular Research Center and Center for Human Genetic Research, Massachusetts General Hospital, Harvard Medical School, Boston, Massachusetts, United States of America; 21 Department of Epidemiology, Johns Hopkins University, Baltimore, Maryland, United States of America; 22 INSERM U1012, Bicêtre, France; 23 University Paris-Sud, Bicêtre, France; 24 AP-HP, Department of Internal Medicine and Infectious Diseases, Bicêtre Hospital, Bicêtre, France; 25 Department of Medical Microbiology, University of Manitoba, Winnipeg, Manitoba, Canada; 26 National Microbiology Laboratory, Winnipeg, Manitoba, Canada; 27 Institute for Immunology & Infectious Diseases, Murdoch University and Pathwest, Perth, Australia; 28 Department of Experimental Immunology, Sanquin Research, Landsteiner Laboratory, and Center for Infectious Diseases and Immunity Amsterdam (CINIMA) at the Academic Medical Center of the University of Amsterdam, Amsterdam, The Netherlands; 29 Institució Catalana de Recerca i Estudis Avançats (ICREA), Barcelona, Spain; 30 Inserm, CESP U1018, University Paris-Sud, UMRS 1018, Faculté de Médecine Paris-Sud; AP-HP, Hopital Bicêtre, Epidemiology and Public Health Service, Le Kremlin Bicêtre, France; 31 Infectious Diseases Service. Hospital Clinic – IDIBAPS, University of Barcelona, Barcelona, Spain; 32 Department of Infectious Diseases, The National University Hospital, Rigshospitalet, Copenhagen, Denmark; 33 Theodosius Dobzhansky Center for Genome Bioinformatics, St. Petersburg State University, St. Petersburg, Russia; 34 Division of Infectious Disease, Brigham and Women's Hospital, Harvard Medical School, Boston, Massachusetts, United States of America; 35 Division of Immunology, Transplantation and Infectious Diseases, Vita-Salute San Raffaele University, School of Medicine & San Raffaele Scientific Institute, Milan, Italy; 36 Genetic Epidemiology Group, Wellcome Trust Sanger Institute, Hinxton, United Kingdom; 37 Non-Communicable Disease Research Group, Department of Public Health and Primary Care, University of Cambridge, Cambridge, United Kingdom; 38 Center for Human Genome Variation, Duke University School of Medicine, Durham, North Carolina, United States of America; 39 INSERM UMRS 945, Paris, France; 40 School of Biology, Georgia Institute of Technology, Atlanta, Georgia, United States of America; 41 Howard Hughes Medical Institute, Chevy Chase, Maryland, United States of America; 42 Infectious Disease Clinical Research Program, Uniformed Services University of the Health Sciences, Bethesda, Maryland, United States of America; 43 Basic Research Laboratory, Molecular Genetic Epidemiology Section, Center for Cancer Research, NCI, SAIC-Frederick, Inc., Frederick National Laboratory, Frederick, Maryland, United States of America; 44 Division of Infectious Diseases, The Feinberg School of Medicine, Northwestern University, Chicago, Illinois, United States of America; 45 Division of Genetics Brigham and Women's Hospital, Harvard Medical School, Boston, Massachusetts, United States of America; 46 Department of Medical Genetics, University Medical Center Utrecht, Utrecht, The Netherlands; 47 Department of Epidemiology, University Medical Center Utrecht, Utrecht, The Netherlands; University College London, United Kingdom

## Abstract

Multiple genome-wide association studies (GWAS) have been performed in HIV-1 infected individuals, identifying common genetic influences on viral control and disease course. Similarly, common genetic correlates of acquisition of HIV-1 after exposure have been interrogated using GWAS, although in generally small samples. Under the auspices of the International Collaboration for the Genomics of HIV, we have combined the genome-wide single nucleotide polymorphism (SNP) data collected by 25 cohorts, studies, or institutions on HIV-1 infected individuals and compared them to carefully matched population-level data sets (a list of all collaborators appears in Note S1 in Text S1). After imputation using the 1,000 Genomes Project reference panel, we tested approximately 8 million common DNA variants (SNPs and indels) for association with HIV-1 acquisition in 6,334 infected patients and 7,247 population samples of European ancestry. Initial association testing identified the SNP rs4418214, the C allele of which is known to tag the HLA-B*57:01 and B*27:05 alleles, as genome-wide significant (p = 3.6×10^−11^). However, restricting analysis to individuals with a known date of seroconversion suggested that this association was due to the frailty bias in studies of lethal diseases. Further analyses including testing recessive genetic models, testing for bulk effects of non-genome-wide significant variants, stratifying by sexual or parenteral transmission risk and testing previously reported associations showed no evidence for genetic influence on HIV-1 acquisition (with the exception of *CCR5Δ32* homozygosity). Thus, these data suggest that genetic influences on HIV acquisition are either rare or have smaller effects than can be detected by this sample size.

## Author Summary

Comparing the frequency differences between common DNA variants in disease-affected cases and in unaffected controls has been successful in uncovering the genetic component of multiple diseases. This approach is most effective when large samples of cases and controls are available. Here we combine information from multiple studies of HIV infected patients, including more than 6,300 HIV+ individuals, with data from 7,200 general population samples of European ancestry to test nearly 8 million common DNA variants for an impact on HIV acquisition. With this large sample we did not observe any single common genetic variant that significantly associated with HIV acquisition. We further tested 22 variants previously identified by smaller studies as influencing HIV acquisition. With the exception of a deletion polymorphism in the *CCR5* gene (*CCR5Δ32*) we found no convincing evidence to support these previous associations. Taken together these data suggest that genetic influences on HIV acquisition are either rare or have smaller effects than can be detected by this sample size.

## Introduction

Variation in infection susceptibility and severity is a hallmark of infectious disease biology. This natural variation can be attributed to a variety of host, pathogen and environmental factors, including host genetics. Several genome-wide association studies (GWAS) of HIV-1 outcomes have been performed primarily to assess the impact of human genetic variation on plasma viral load and/or disease progression [1], [2], [3], [4], [5], [6], [7], [8], [9], [10], [11]. These studies have confirmed the key role of major histocompatibility complex (MHC) polymorphisms in HIV-1 control, with a minor impact of variants in the *CCR5* gene region.

A smaller number of GWAS have also investigated host genetic influences on HIV-1 acquisition using samples of individuals with known or presumed exposure to an HIV-1 infected source [12], [13], [14], [15], [16]. With the exception of *CCR5Δ32* homozygosity (known to explain a proportion of HIV-1 resistance in Europeans [17]), no reproducible associations with increased or reduced HIV-1 acquisition have been observed. Additionally, several variants reported to influence HIV-1 acquisition by candidate gene studies have either failed to be replicated or lacked sufficient investigation as to be considered confirmed.

We here describe a large study of human genetic determinants of HIV-1 acquisition, performed under the auspices of the International Collaboration for the Genomics of HIV, a collaborative research effort bringing together the HIV-1 host genetics community. By collecting for the first time all available genome-wide single nucleotide polymorphism (SNP) data on HIV-1 infected individuals and comparing them with population-level control data sets we sought to uncover common genetic markers that influence HIV-1 acquisition.

## Results

### Association testing and meta-analysis

Genome-wide genotype data were collected from 25 cohort studies and clinical centers (listed at the end of the paper and in Note S1 in Text S1). We obtained a data set of 11,860 HIV-1 infected individuals genotyped at multiple centers using several platforms (Table S1 in Text S1). The present analysis focused on the subset of these individuals that are of European ancestry as assessed by principal components (PCs) analysis (see methods). For two of the genotyping centers, matched HIV-1 uninfected controls were available. For the remaining samples, large population-level control data sets were accessed from the Illumina Genotype Control Database (www.illumina.com) and the Myocardial Infarction Genetics (MIGen) Consortium (genotyped using the Affymetrix 6.0 platform) [18]. Sample-level quality control and case-control matching (Figure S1 in Text S1) resulted in six non-overlapping data sets including 6,334 HIV-1 infected cases and 7,247 controls (Table S1 in Text S1). After imputation, each variant was individually tested for association with HIV-1 status by logistic regression including PCs to correct for residual population structure, under additive and recessive genetic models. Association results were then combined across data sets.

**Figure 1:**
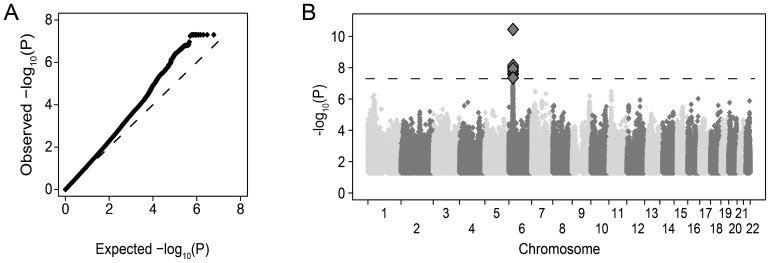
Association results for approximately 8 million common DNA variants tested for an impact on HIV-1 acquisition. A) Quantile-quantile plot of association results after meta-analysis across the six groups. For each variant tested, the observed −log_10_ p-value is plotted against the null expectation (dashed line). P-values lower than 5×10^−8^ are truncated for visual effect. B) Manhattan plot of association results where each variant is plotted by genomic position (x-axis) and −log_10_ p-value (y-axis). Only variants in the MHC region on chromosome 6 have p-values below genome-wide significance (p<5×10^−8^ dashed line, large diamonds).

**Figure 2:**
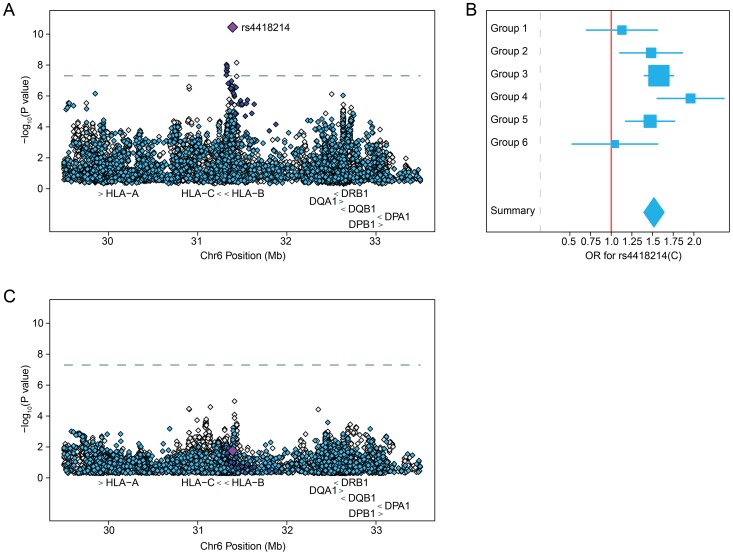
Common DNA variants within the MHC region that are associated with HIV-1 acquisition comparing 6,334 HIV-1 infected patients to 7,247 population controls are driven by HIV-1 controllers and not maintained when restricting to patients with known dates of seroconversion. A) Regional association plot of the locus containing genome-wide significant SNPs after meta-analysis. The signal of association is centered on the *HLA-B/HLA-C* genes. The association result for the top SNP, rs4418214, is indicated by the purple diamond, with dark blue indicating SNPs in high LD (r^2^>0.8), light blue indicating moderate LD (r^2^ between 0.2 and 0.8) and grey indicating low or no LD (r^2^<0.2) with rs4418214. The dashed line indicates genome-wide significance (p<5×10^−8^). The location of classical class I and class II HLA genes (green arrows) is given as reference. B) Forest plot of effect estimates for the C allele at rs4418214 with 95% confidence intervals per group (box and whiskers) and after meta-analysis (diamond). The majority of the association signal is contributed by Groups 3 and 4, which are enriched for HIV-1 controllers. C) Regional association plot of the same variants as in A) but restricting analysis to include only individuals with a known date of seroconversion to limit frailty bias.

Restricting to variants observed in all six data sets with >1% frequency and a minimum imputation quality of 0.8 in at least 2 groups, approximately 8×10^6^ common variants (SNPs and indels) were tested. The overall distribution of p-values was highly consistent with the null hypothesis (λ_1000_ = 1.01) suggesting that the matching strategy was successful in minimizing inflation (Figure 1a). We observed 11 SNPs with combined evidence for association passing the genome-wide significance threshold (p<5×10^−8^, Figure 1b) under an additive genetic model. All genome-wide significant SNPs were located in the MHC region, centered on the class I HLA genes *HLA-B/HLA-C* (Figure 2a and Table S2 in Text S1). The top SNP, rs4418214 (p = 3.6×10^−11^, odds ratio (OR) for the C allele = 1.52) has previously been associated with control of HIV-1 viral load [8], with the C allele tagging the classical *HLA-B* alleles 57:01 and 27:05, both known to associate with lower viral load and longer survival after infection. Analysis assuming a recessive genetic model did not identify any genome-wide significant associations (data not shown).

### Exploration of top associations

Since variation in the HLA region is well known to impact rate of HIV-1 disease progression and not acquisition, we sought to better understand the observed associations at this locus. Due to their shorter survival time, patients with rapid disease progression are underrepresented in seroprevalent cohorts, while individuals with prolonged disease-free survival times are more likely to be included, leading to an enrichment of factors that protect against disease progression in such populations. Additionally, some of the cohorts accessed for this analysis specifically recruited long-term non-progressors (LTNPs, Groups 2, 3 and 4). Inspection of the effect estimates at the top SNP (rs4418214) per data set showed that the majority of the association signal was driven by groups specifically enriched for LTNPs (Figure 2b) suggesting a possible frailty bias in the overall results.

To assess the potential contribution of frailty bias, we ran association testing as previously but restricting the case population to 2,173 individuals with a known date of seroconversion that were not enrolled in LTNP cohorts. Association testing in this sample showed no variants passing the genome-wide significance threshold. Additionally, rs4418214 dramatically dropped in strength of association to p = 0.02, with all other previously genome-wide significant SNPs suffering a similar loss in association strength (Figure 2c and Table S2 in Text S1). In order to address whether this loss of association signal could be due to the reduced size of the case population rather than frailty bias, we performed a sensitivity analysis where we tested for association at rs4418214 restricting the HIV+ cases to 2,173 individuals randomly selected from the full case sample. We repeated this procedure 1,000 times and compared the p-value from the random case selection to that obtained when restricting to seroconverters. Of these 1,000 tests, only one resulted in a loss of association signal that was similar to what was observed when restricting to seroconverters (Figure S2 in Text S1). This suggests that the signal observed in the full acquisition analysis is most likely due to frailty bias.

### Polygenic analysis

**Figure 3:**
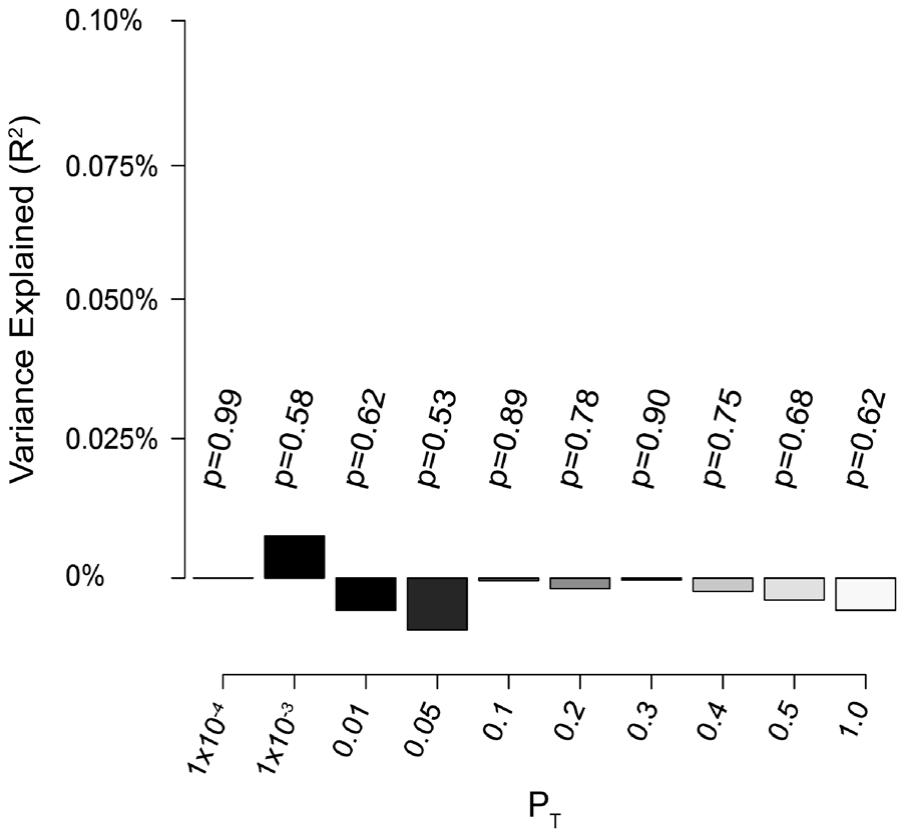
Analysis of bulk SNP effects shows no evidence for enrichment of association signal across data sets. LD pruned SNP sets falling below various p-value thresholds (grey shades, x-axis) were selected based on association results calculated in five of six groups (discovery set). Per individual scores were calculated in a non-overlapping test set (Group 3) by summing the beta-weighted dosage of all SNPs in that set. Model p-value (listed above bars) and variance explained (using Nagelkerke's pseudo R^2^, y-axis) were calculated by regressing phenotype on per individual score using logistic regression.

Previous studies in large cohorts have shown that multiple genetic variants with small effect sizes that contribute to complex traits, but fall below the genome-wide significance threshold, can be detected by examining the consistency of their combined effects across studies [19]. We sought to test for evidence of such polygenic inheritance in our study population. To do this (and to avoid overfitting), we split our sample into a discovery set (Groups 1,2,4,5 and 6) and a test set (Group 3) and performed genome-wide association testing and meta-analysis on the discovery set. Based on these results, we generated sets of high-quality SNPs (minor allele frequency >0.1, imputation accuracy >0.9) in relative linkage equilibrium (r2<0.1, informed by p-value in the discovery set, see methods) falling below various p-value thresholds (P_T_). Scores were then generated for all individuals in Group 3 by summing the weighted genotype dosage (using the log odds ratio from the discovery set as weights) of all SNPs below a given P_T_. Phenotype was then regressed on this score using logistic regression including covariates. We assessed both the significance of the score and the phenotypic variance explained (using Nagelkerke's pseudo-R^2^ [20]). We did not observe a significant association between the calculated score and phenotype in the discovery set at any P_T_ (Figure 3). This further suggests that effects of common variants on HIV-1 acquisition detectable by this study design are negligible.

### Analysis by transmission risk

Since different modes of HIV-1 transmission may be influenced by different host factors, we further investigated if genetic variants may contribute to enhanced HIV-1 acquisition within transmission risk sub-groups. We stratified the study population by reported risk groups that were either primarily sexual (homosexual and heterosexual, n = 3,311) or parenteral (injection drug use and transfusion, n = 1,046). Association results in these sub-groups were consistent with those observed in the full set with no genome-wide significant signals detected (data not shown).

### Association testing of variants previously reported to influence HIV-1 acquisition

With the exception of *CCR5Δ32* (addressed in the next section), many variants reported to influence HIV-1 acquisition have remained unconfirmed. We sought to assess the evidence for association of 22 variants previously reported to influence HIV-1 acquisition in this sample. All 22 of these variants could be measured in this sample either through direct genotyping or imputation. Of these, only one variant (rs1800872) showed nominal significance (p<0.05, Table 1) although it did not survive correction for the number of variants tested (p>2.5×10^−3^). Thus, none of the previously reported associations can be considered confirmed in this large sample.

**Table 1:** Results for 22 SNPs previously reported to affect HIV-1 acquisition sorted by reported effect and genomic location.

SNP	CHR	BP (hg19)	A1	A2	Frequency HIV+	Frequency HIV−	OR	SE	P	Gene	Reported effect on acquisition	Reference
rs1800872	1	206946407	T	G	0.245	0.232	1.08	0.030	0.01	*IL10*	Increased	[34]
rs3732378	3	39307162	A	G	0.163	0.164	0.97	0.035	0.35	*CX3CR1*	Increased	[35]
rs3732379	3	39307256	T	C	0.279	0.282	0.98	0.028	0.46	*CX3CR1*	Increased	[35]
rs6850	7	44836314	G	A	0.123	0.133	0.94	0.039	0.09	*PPIA*	Increased	[36]
rs754618	10	44886206	T	C	0.311	0.304	1.01	0.028	0.73	*CXCL12*	Increased	[37]
rs1946518	11	112035458	G	T	0.590	0.592	0.98	0.026	0.49	*IL18*	Increased	[38]
rs2280789	17	34207003	G	A	0.136	0.134	1.04	0.038	0.30	*CCL5*	Increased	[39]
rs2280788	17	34207405	C	G	0.022	0.023	0.90	0.088	0.25	*CCL5*	Increased	[39]
rs2107538	17	34207780	T	C	0.183	0.180	1.02	0.034	0.49	*CCL5*	Increased	[39]
rs2549782	5	96231000	T	G	0.477	0.477	1.00	0.026	0.94	*ERAP2*	Decreased	[40]
rs2070729	5	131819921	A	C	0.428	0.426	1.02	0.026	0.50	*IRF1*	Decreased	[41]
rs2070721	5	131825842	G	T	0.427	0.426	1.02	0.026	0.50	*IRF1*	Decreased	[41]
rs6996198	8	65463442	T	C	0.159	0.167	0.97	0.035	0.46	*CYP7B1*	Decreased	[42]
rs1552896	9	14841387	G	C	0.227	0.227	1.01	0.032	0.77	*FREM1*	Decreased	[15]
rs1801157	10	44868257	T	C	0.200	0.209	0.97	0.032	0.36	*CXCL12*	Decreased	[37]
rs10838525	11	5701001	T	C	0.357	0.355	1.00	0.027	0.95	*TRIM5*	Decreased	[43]
rs3740996	11	5701281	A	G	0.113	0.117	0.93	0.040	0.05	*TRIM5*	Decreased	[44]
rs1024611	17	32579788	G	A	0.267	0.277	0.95	0.029	0.08	*CCL2*	Decreased	[45]
rs1024610	17	32580231	T	A	0.200	0.205	0.97	0.032	0.31	*CCL2*	Decreased	[46]
rs2857657	17	32583132	G	C	0.196	0.200	0.97	0.032	0.32	*CCL2*	Decreased	[46]
rs4795895	17	32611446	A	G	0.193	0.196	0.97	0.032	0.40	*CCL11*	Decreased	[46]
rs1719134	17	34416946	A	G	0.240	0.231	1.05	0.031	0.13	*CCL3*	Decreased	[45]

Reported effects correspond to the A1 allele.

Frequency and odds ratio (OR) are calculated for the A1 allele with an OR>1 indicating a higher frequency of A1 in the HIV-1 infected sample.

### Power for variant detection

Parameters required for determining power for variant detection, specifically the trait prevalence and the level of enrichment of enhanced HIV-1 acquisition, are difficult to estimate given this study design. Thus, we sought to determine the extent to which we could detect known genetic influences on HIV-1 acquisition in this sample by assessing the depletion of *CCR5Δ32* homozygosity in the HIV-1 infected sample. Although this variant is not captured by commercial arrays (and is not included in the 1,000 Genomes Project reference panel), genotypes of the deletion were available for a majority of the HIV-1 infected individuals (n = 4,854). As expected, we observed very few Δ32/Δ32 homozygous individuals in this sample (n = 4) and a large deviation from Hardy-Weinberg equilibrium (Table S3 in Text S1).

To assess the association strength of this variant, we used a subset of our sample with available *CCR5Δ32* genotypes to build a reference panel, which was then used for imputation of *CCR5Δ32* in both cases and controls (see methods). Overall the imputation accuracy was acceptable (average information score = 0.82) and we observed good correspondence between typed and imputed dosage (Figure S3 in Text S1). Using a recessive genetic model, we observed a genome-wide significant association between *CCR5Δ32* homozygosity and HIV-1 acquisition (p = 5×10^−9^, OR = 0.2). No impact on HIV-1 acquisition was observed under any other genetic model.

To address whether the association signal at *CCR5Δ32* was subject to the same frailty bias as the MHC SNPs, we next tested for association between *CCR5Δ32* and HIV acquisition restricting only to the 2,173 HIV+ individuals with known dates of seroconversion. Using these individuals, *CCR5Δ32* remains strongly associated (p = 1×10^−6^ for the recessive model), suggesting that the observed association statistic in the full set is not simply due to frailty bias. This demonstrates that, despite an inability to precisely estimate power, other variants of similar or somewhat weaker effect could also have been detected in this sample.

## Discussion

By assembling a large collaborative network of cohorts and institutions involved in HIV-1 host genetic studies we sought to test for common genetic polymorphisms that influence HIV-1 acquisition. Through this network, we were able to combine genome-wide SNP data on over 6,300 HIV-1 infected patients of European ancestry. In order to maximize power, we further accessed large population-level genotype data sets to use as controls. Where necessary, case/control samples were iteratively matched to limit inflation in the test statistic due to platform or cohort effects. Genome-wide imputation using the 1,000 Genomes Project CEU sample as a reference panel resulted in a set of approximately 8×10^6^ high-quality variants that were tested for association with HIV-1 acquisition. We observed 11 variants that passed the genome-wide significance threshold, all located in the MHC region. Imputation and association testing of the *CCR5Δ32* polymorphism demonstrated that this sample size and study design are appropriate to detect strong associations that impact HIV-1 acquisition.

The fact that the top association in the full analysis (rs4418214) is a tag SNP for HLA-B*57:01 and 27:05 highlights the frailty bias inherent to studies of diseases with high mortality rates. *HLA-B* alleles have been associated with reduced HIV-1 transmission in heterosexual couples [21], likely due to the effects of HLA-B on HIV-1 viral load, which decreases infectiousness. To further explore the possibility that *HLA-B* alleles are also associated with HIV-1 acquisition, we ran an analysis restricting the case population to the 2,173 individuals with a known date of seroconversion, assuming that cohorts of patients recruited soon after HIV-1 acquisition are less likely to suffer from frailty bias. This analysis resulted in an almost complete loss of signal at rs4418214 that is unlikely to be due to the reduction in size of the case population. Thus, the most parsimonious explanation for the association result in the HLA class I region is that it reflects an enrichment of alleles that protect against disease progression (hence survival) rather than increasing acquisition.

Under ideal circumstances, this sample size provides approximately 80% power to detect a common variant (MAF = 0.1) with genotypic relative risk of 1.3 at genome-wide significance. However, we recognize that the present study design allows for a proportion of the sample to be misclassified (i.e. individuals at average or low susceptibility to HIV-1 infection included as cases) which can reduce power [22]. Nevertheless, even under assumptions including a large proportion of controls in the case group, this sample size is suitable to discover large effect variants (GRR>3, Figure S4 in Text S1). This is further evidenced by our ability to detect the known effect of *CCR5Δ32* homozygosity on HIV-1 acquisition in this sample, even given imperfect imputation.

Additionally, the lack of enrichment of the control population for individuals with proven or suspected resistance against HIV-1 infection may also influence power [23]. However, in line with our results, GWAS looking at HIV-1 acquisition in mother-to-child transmission pairs [12], discordant couples [13], areas of heightened prevalence [14] and in hemophiliacs exposed to potentially contaminated blood products [16] (although much smaller than the present study) have been similarly unable to discover novel associations.

This large study population is useful for attempting to replicate previous associations, particularly with genetic variants thought to reduce HIV-1 acquisition, as they would be depleted in infected individuals. None of the 22 previously reported variants tested in this sample were associated with HIV-1 acquisition after correcting for multiple tests. This lack of replication is consistent with other, smaller GWAS of this phenotype [14]. These data suggest that many or all of these variants do not appreciably impact HIV-1 acquisition. Thus, evidence is mounting that common polymorphisms affecting acquisition are either very difficult to detect (perhaps due to weak effects) or absent, with the exception of *CCR5Δ32* homozygosity.

The early observation that *CCR5Δ32* influences both acquisition (when homozygous) and disease progression (when heterozygous) suggested shared biology between these phenotypes. However, this proved not to be a generalizable observation since variation at other loci, such as HLA class I and KIR, associate with disease progression but are not generally believed to modulate acquisition. Mechanisms mediating acquisition i.e. permissiveness to HIV upon parenteral or mucosal exposure, likely involve cellular targets and innate immune factors that play none or a limited role in disease progression. On the other hand, mediators of host tolerance (as defined by [24]) and of acquired immunity are only expected to exert their effects after infection has been established.

Although this study focuses on the host genetics of HIV-1 acquisition, it is possible that the extensive variation in HIV-1 genotype also plays a role in determining susceptibility. This notion is supported by the observation that amino acid changes, generally in response to host HLA pressure, result in decreased viral fitness (reviewed in [25]). However, defining viral variants that limit or enhance infection would require large-scale epidemiological investigations in HIV-1 endemic areas.

Despite the large sample size and comprehensive genotype information obtained through imputation, this study is still limited to analysis of common variation with detectable effects present in European samples. Thus, we cannot rule out whether multiple common variants of small effect, population-specific variants or rare variants exist that influence HIV-1 acquisition. Of particular note, in light of the well-known effect of *CCR5Δ32* on HIV acquisition, is the inability to comprehensively test structural variation using array-based genotyping platforms. Although SNPs contained on commercial arrays have been shown to tag a large proportion of common structural variants [26] it is still possible that unobserved or poorly tagged structural variants contribute to HIV acquisition. Detection of these types of effects will require large-scale sequencing efforts, preferably in samples with known levels of exposure to HIV-1.

## Materials and Methods

### Ethics statement

Ethical approval for this study was obtained from institutional review boards at each of the Cohorts, Studies and Centers listed at the end of the manuscript. All subjects provided written informed consent.

### Sample collection, genotyping and quality control

The International Collaboration for the Genomics of HIV was established as a platform to combine all available genome-wide SNP data sets obtained on HIV-1 infected individuals worldwide. Patient material was collected at multiple clinical centers across North America, Europe, Australia and Africa (a list of contributing cohort studies and centers is given at the end of the paper). Genotypes for uninfected control individuals were obtained directly from three of the participating centers (GRIV, ACS, CHAVI) and from the Illumina genotype control database (www.illumina.com/icontroldb) and the Myocardial Infarction Genetics Consortium (MIGen) (NIH NCBI dbGaP Study Accession: phs000294.v1.p1) [18]. Each data set was subject to quality control procedures performed prior to centralization of all data for the combined analysis. However, to ensure consistency, all data were subject to further quality control once submitted. Per data set, samples with high missingness (<95% of sites successfully genotyped) and high heterozygosity (inbreeding coefficient >0.1) were removed. Ancestry was determined using EIGENSTRAT to project sample data onto the HapMap III reference panel. For the present analysis, only individuals clustering with the CEU/TSI subset were retained. To remove samples genotyped by multiple centers (and those with high relatedness) we performed identity-by-state analysis taking the intersection of SNPs across all genotyping platforms, using PLINK version 1.07 [27]. In the case of duplicates, the sample contributing the larger number of genotyped SNPs was retained. We further filtered out individuals with relatedness higher than 0.125, adopting a strategy to maximize sample retention. After sample removal, SNPs with high missingness (>2%), low minor allele frequency (<1%) or that were out of Hardy-Weinberg equilibrium (p<1×10^−6^) were removed.

### Case/control matching

To limit bias introduced due to the majority of the control samples being genotyped separately from cases we used a 2-stage case/control matching strategy. In the first round, cases and controls were matched by platform and geographic origin. This resulted in four clusters; The Netherlands (Illumina, Group 1), France (Illumina, Group 2), North America and non-Dutch/non-French European (Illumina, Groups 3 and 4), North America and non-Dutch/non-French European (Affymetrix, Groups 5 and 6). To test the success of this method at controlling inflation, we ran association testing on all genotyped SNPs including the top PCs as covariates per cluster and assessed lambda (Figure S1 in Text S1). For samples ascertained from France and The Netherlands, this was sufficient to control inflation in the test statistic (λ∼1, Figures S1a–d in Text S1). For the remaining two clusters, we plotted each sample based on their coordinates across the top two PCs and split each cluster into two sub-clusters based on these coordinates. Sub-clusters then underwent either 1∶3 or 1∶1 case/control matching using Euclidean distance across the top 10 PCs (with the top PC given twice the weight of the others). Samples were removed if no suitable match could be identified. This strategy proved sufficient to control inflation in these remaining clusters (Figures S1e–l in Text S1).

### Imputation and association testing

After sample matching and per group quality control, unobserved SNP genotypes were imputed using the 1,000 Genomes Project Phase I release integrated SNPs and indels (March 2012). Two teams from this Collaboration performed the analysis independently using different tools. The first team used BEAGLE [28], the second team used the pipeline IMPUTE2, SNPTEST and META [29], [30] with a pre phasing step using ShaPEIT [31]. Per group, phenotype was regressed on genotype dosage including population covariates calculated by EIGENSTRAT to control for residual structure under both additive and recessive genetic models. Association results were then combined using inverse-variance weighted meta-analysis including a covariate to correct for group-specific effects. The results obtained by each team were compared for cross-validation and found to be highly consistent (Figure S5 in Text S1). SNPs were considered associated if the combined p-value was below the accepted level of genome-wide significance (p<5×10^−8^).

### Polygenic analysis

We performed analysis to test for evidence of polygenic effects using five of the six groups as a discovery set and Group 3 (the largest single group) as the test set. To build a SNP set we first filtered out all SNPs with low minor allele frequency (MAF<0.1) and low imputation quality (R^2^<0.9) and removed the MHC region. We then performed LD pruning informed by the p-value calculated in the discovery set such that the SNP with the lowest p-value was selected and all other SNPs in LD (r^2^>0.1) were removed. The SNP with the lowest remaining p-value was then selected and again all other SNPs in LD were removed. This procedure was repeated until no remaining SNPs fell below the selected P_T_. In the test set, per individual scores were generated by summing the dosage of all SNPs in a set weighted by the effect size (beta) calculated in the discovery set. We then regressed phenotype on this score using logistic regression including top PCs. SNP set pruning was performed using PLINK version 1.07 [27], logistic regression, calculation of variance explained and results visualization was performed using R version 2.12 (www.r-project.org) and the Design package [32].

### Testing previous associations

A list of SNPs previously reported to associate with HIV-1 acquisition was taken from Petrovski et al [14] and updated to include recently reported associations. All SNPs were either directly genotyped or imputed, and tested in the same logistic regression/meta-analysis framework as all other variants.

### Imputation and association testing of *CCR5Δ32*

*CCR5Δ32* genotypes were obtained by individual cohorts using either Sequenom genotyping, PCR or direct sequencing as described in the original publications. Since genotype of this deletion was not available in the control populations we used a subset of the HIV+ sample with both genome-wide genotypes and *CCR5Δ32* types as a reference panel for imputation. For this, we used the subset typed on the Illumina 1M platform (n = 1,100) to maximize SNP coverage. Additionally, we included 383 non-overlapping individuals with known *CCR5Δ32* genotype from a recent GWAS in hemophiliacs [16]. Phasing of the reference panel and imputation was performed using ShaPEIT [31] and IMPUTE2 [29], [30]. We imputed *CCR5Δ32* genotype in both cases and controls using a *leave-one-out* strategy such that, if an individual was present in both the reference and test sample, their genotype information was removed from the reference panel and imputation was carried out using the remaining samples as reference. Association was tested under a recessive model and assuming an additive or heterozygous advantage model.

### Estimating power for variant detection

Power for variant detection was estimated over a wide range of possible proportions of controls being misclassified as cases (Figure S4 in Text S1). Calculations were made under an additive genetic model assuming a risk variant of 10% frequency for a study of 6,300 cases and 7,200 controls at genome-wide significance (p<5×10^−8^). Calculations were performed using PAWE-3D [22], [33].

### Cohorts, studies, and centers participating in the International Collaboration for the Genomics of HIV

1. The AIDS clinical Trial Group (ACTG) in the USA
2. The AIDS Linked to the IntraVenous Experience (ALIVE) Cohort in Baltimore, USA
3. The Amsterdam Cohort Studies on HIV infection and AIDS (ACS) in the Netherlands
4. The ANRS CO18 in France
5. The ANRS PRIMO Cohort in France
6. The Center for HIV/AIDS Vaccine Immunology (CHAVI) in the USA
7. The Danish HIV Cohort Study in Denmark
8. The Genetic and Immunological Studies of European and African HIV-1+ Long Term Non-Progressors (GISHEAL) Study, in France and Italy
9. The GRIV Cohort in France
10. The Hemophilia Growth and Development Study (HGDS) in the USA
11. The Hospital Clinic-IDIBAPS Acute/Recent HIV-1 Infection cohort in Barcelona, Spain
12. The Icona Foundation Study in Italy
13. The International HIV Controllers Study in Boston, USA
14. The IrsiCaixa Foundation Acute/Recent HIV-1 Infection cohort in Barcelona, Spain
15. The Modena Cohort in Modena, Italy
16. The Multicenter AIDS Cohort Study (MACS), in Baltimore, Chicago, Pittsburgh and Los Angeles, USA
17. The Multicenter Hemophilia Cohort Studies (MHCS)
18. The NCI Laboratory of Genomic Diversity in Frederick, USA
19. The Pumwani Sex Workers Cohort in Nairobi, Kenya, and Winnipeg, Canada
20. The San Francisco City Clinic Cohort (SFCCC) in San Francisco, USA
21. The Sanger RCC Study in Oxford, UK, and in Uganda
22. The Swiss HIV Cohort Study (SHCS), in Switzerland
23. The US military HIV Natural History Study (NHS)
24. The Wellcome Trust Case Control Consortium (WTCCC3) study of the genetics of host control of HIV-1 infection in the Gambia
25. The West Australian HIV cohort Study

## Supporting Information

Text S1 Includes Note S1: the cohorts and individuals contributing to the International Consortium for the Genomics of HIV, Tables S1, S2, S3, Figures S1, S2, S3, S4, S5 and supplementary references.
(DOC)
