# Supplementary material for: Association Study of Common Genetic Variants and HIV-1 Acquisition in 6,300 Infected Cases and 7,200 Controls
Source: PLoS Pathog. 2013 Jul 25;9(7):e1003515. doi: 10.1371/journal.ppat.1003515 (PMC3723635; doi:10.1371/journal.ppat.1003515)
Supplement: Text S1 — Includes Note S1: the cohorts and individuals contributing to the International Consortium for the Genomics of HIV, Tables S1, S2, S3, Figures S1, S2, S3, S4, S5 and supplementary references. (DOC) [file ppat.1003515.s001.doc]

**Text S1: Supplementary materials**

Association Study of Common Genetic Variants and HIV-1 Acquisition in 6,300 Infected Cases and 7,200 Controls

**Table S1** Characteristics of matched case/control sample groups used for association testing. Lambda was calculated based on association results after imputation using HapMap III as the reference panel and scaled to 1,000 cases/1,000 controls.

| **Group** | **Cases** | **Controls** | **Matching strategy** | **λ_1000_** | **Platforms** |
| --- | --- | --- | --- | --- | --- |
| Group 1 | 401 | 998 | Matched controls provided | 1.04 | Illumina 300, Illumina 1M |
| Group 2 | 850 | 672 | Matched controls provided | 1.07 | Ilumina 300 |
| Group 3 | 2759 | 2759 | 1:1 case/control | 1.03 | Illumina 550, Illumina 650, Illumina 1M |
| Group 4 | 986 | 513 | 2:1 case/control | 1.15 | Illumina 550, Illumina 650, Illumina 1M |
| Group 5 | 1026 | 1190 | 1:1 case/control | 1.03 | Affymetrix 6.0 |
| Group 6 | 312 | 1115 | 1:3 case/control | 1.06 | Affymetrix 6.0 |

**Table S2** Association results of genome-wide significant SNPs in the full sample and after restricting to seroconverters. Odds ratio (OR) is calculated for A1, which is set as the allele at higher frequency in HIV+ compared to population controls

|  |  |  | **Full Sample** | | **Seroconverters** | |
| --- | --- | --- | --- | --- | --- | --- |
| **SNP** | **A1** | **A2** | **OR** | **P-value** | **OR** | **P-value** |
| rs4418214 | C | T | 1.52 | 3.6E-11 | 1.24 | 1.8E-2 |
| rs2523674 | A | G | 1.18 | 7.1E-9 | 1.16 | 3.0E-4 |
| rs41557415 | G | A | 1.51 | 9.4E-9 | 1.19 | 9.2E-2 |
| rs112515516 | G | A | 1.53 | 1.2E-8 | 1.27 | 2.5E-2 |
| rs76518703 | G | A | 1.51 | 1.6E-8 | 1.26 | 3.2E-2 |
| rs41543314 | G | A | 1.50 | 2.3E-8 | 1.18 | 1.1E-1 |
| rs3094550 | A | C | 1.22 | 2.6E-8 | 1.24 | 1.5E-5 |
| rs3094551 | G | A | 1.21 | 2.7E-8 | 1.24 | 1.5E-5 |
| rs113688927 | G | A | 1.52 | 2.7E-8 | 1.26 | 3.5E-2 |
| rs1057387 | G | T | 1.51 | 4.4E-8 | 1.18 | 1.2E-1 |
| rs1057151 | C | T | 1.51 | 4.4E-8 | 1.18 | 1.2E-1 |

**Table S3** Frequency of the *CCR5Δ32* polymorphism in a majority subset of HIV-1 infected individuals included in the GWAS

| **Sample group** | **Freq** | **N** | **Hetero-zygotes** | **Homo-zygotes** | **Expected homozygotes*** |
| --- | --- | --- | --- | --- | --- |
| Group 1 | 0.102 | 397 | 81 | 0 | 4 |
| Group 2 | 0.092 | 535 | 98 | 0 | 4 |
| Group 3 | 0.102 | 2641 | 531 | 3 | 27 |
| Group 4 | 0.111 | 765 | 166 | 1 | 9 |
| Group 5 | 0.096 | 423 | 81 | 0 | 4 |
| Group 6 | 0.113 | 93 | 21 | 0 | 1 |
| Total | 0.102 | 4854 | 978 | 4 | 49 |

* Assuming Hardy-Weinberg equilibrium given the observed allele frequency


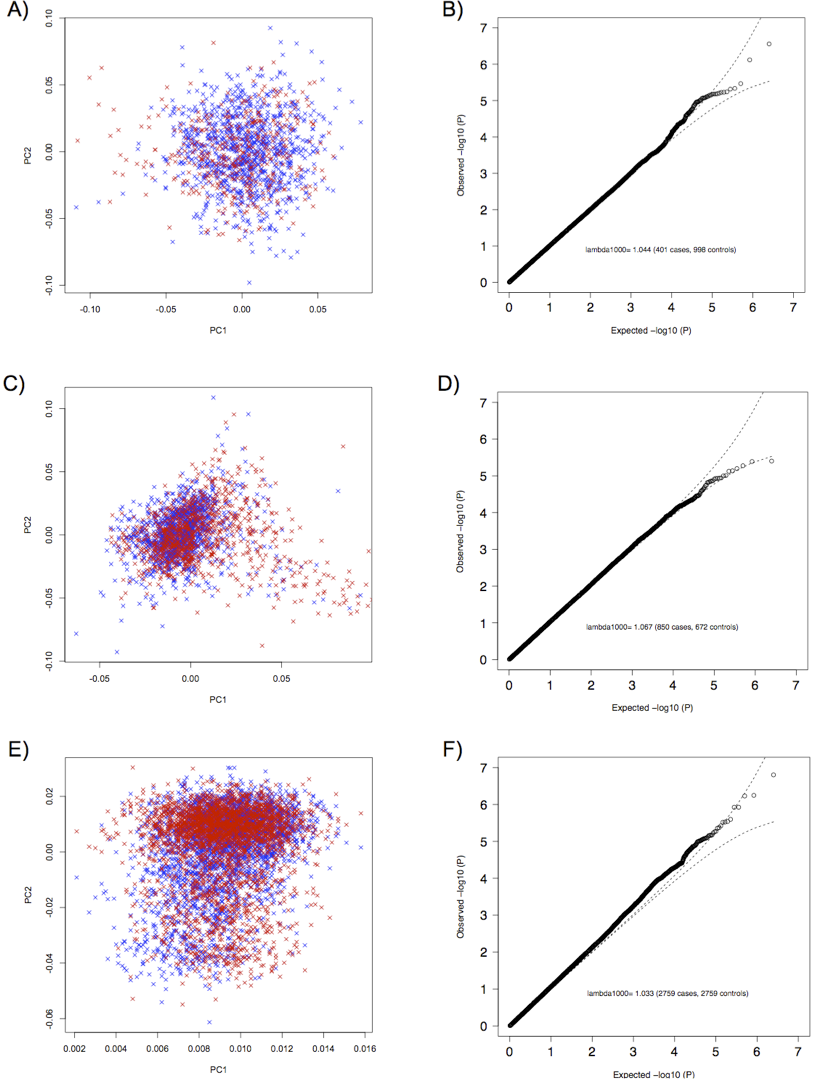


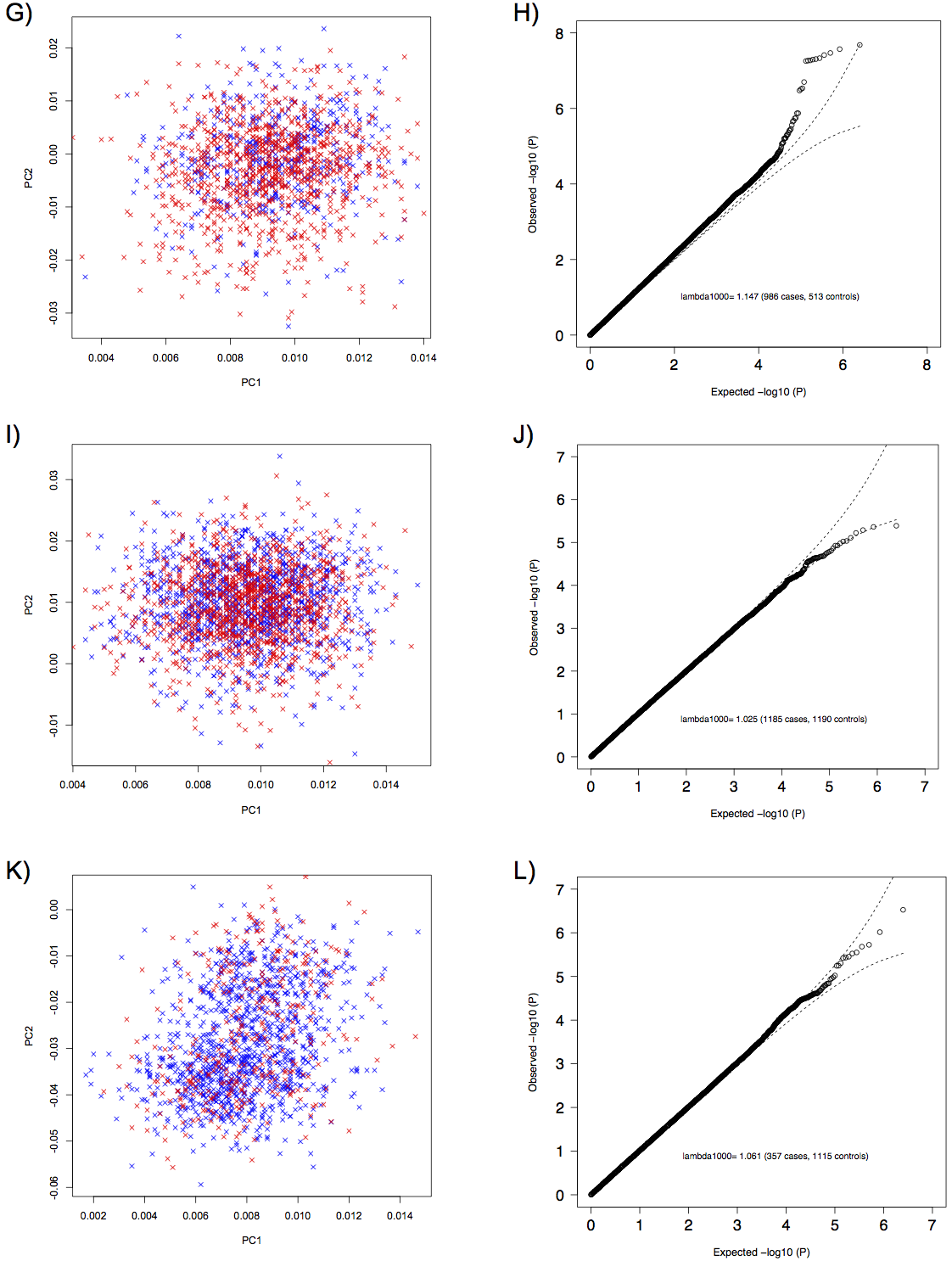


**Figure S1:** Case/control matching and assessment of inflation. Samples were divided into six groups. For Groups 1 and 2 HIV- control individuals were obtained from the same populations as the HIV+ cases and genotyped at the same centers. Two remaining clusters (one typed on Illumina platforms, the other using Affymetrix) were matched to publicly available control data sets genotyped using chips from the same companies. These clusters were further subdivided based on top PCs and underwent case/control matching using a Euclidean distance procedure. A, C, E, G, I and K show all cases (red) and controls (blue) plotted across the top 2 principal components (PC1, PC2) calculated by Eigenstrat for all groups. B, D, F, H, J and L show corresponding QQ plots of observed –log_10_ p-values vs the null expectation with lambda (corrected to 1,000 cases/1,000 controls) calculated across all p-values

**Figure S2:** Distribution of association p-values (-log_10_ transformed) at rs4418214 after 1,000 repetitions of restricting the HIV+ case population to 2,173 individuals randomly selected from the full HIV+ sample. The proportion of repetitions where a similar or greater p-value was detected in the random draw compared to what is seen when restricting to seroconverters falls to the left of the dashed line (0.1% of repetitions).

**Figure S3**: Comparison of genotype and imputed dosage for *CCR5Δ32* in the HIV infected sample. Dosage after imputation was compared with *CCR5* genotype that was directly assessed in 4,854 HIV infected individuals. The overall correspondence is high (imputation quality = 0.82), however Δ32/ Δ32 homozygosity is overestimated. Jitter along the x-axis is added to allow visualization of density.

**Figure S4:** Power estimates accounting for various proportions of controls being misclassified as cases. Power for variant detection was calculated for various genotypic relative risks (blue=1.3, red=2, green=3, purple=4, orange =5) over a wide range of possible proportions of controls being misclassified as cases (x-axis). Calculations were made under an additive genetic model assuming a risk variant of 10% frequency for a study of 6,300 cases and 7,200 controls at genome-wide significance (p<5x10^-8^). The dashed line represents approximately 80% power. Calculations were performed using PAWE-3D .


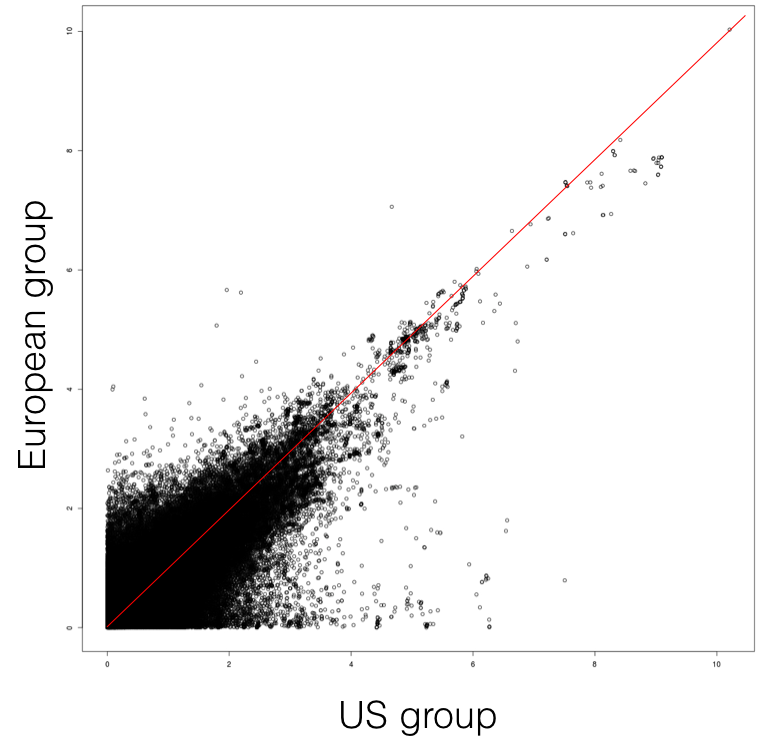


**Figure S5**: Comparison of p-values calculated at parallel analysis sites. Association testing was performed independently at two centers using similar, but not identical, methods for imputation. Plotted are the –log10 p-values calculated at both sites. Overall the results are highly consistent (particularly for the strongest signals).

**Supplementary references:**

1. Gordon D, Finch SJ, Nothnagel M, Ott J (2002) Power and sample size calculations for case-control genetic association tests when errors are present: application to single nucleotide polymorphisms. Human heredity 54: 22-33.

2. Gordon D, Haynes C, Blumenfeld J, Finch SJ (2005) PAWE-3D: visualizing power for association with error in case-control genetic studies of complex traits. Bioinformatics 21: 3935-3937.

**Note S1:**

**Cohorts and Individuals contributing to the International Consortium for the Genomics of HIV**

***The AIDS Clinical Trials Group (ACTG)***

DESCRIPTION:

The AIDS Clinical Trials Group (ACTG) was initially established in 1987 to broaden the scope of the AIDS research effort of the US National Institute of Allergy and Infectious Diseases (NIAID). The ACTG established and supports the largest Network of expert clinical and translational investigators and therapeutic clinical trials units in the world, including sites in resource-limited countries. These investigators and units serve as the major resource for HIV/AIDS research, treatment, care, and training/education in their communities.

MEMBERS:

Eric S. Daar, Roy M. Gulick, David W. Haas, Richard Haubrich, Daniel R. Kuritzkes, Heather J. Ribaudo, Sharon Riddler, Gregory K. Robbins, Paul E. Sax, Robert W. Shafer, Cecilia M. Shikuma

FUNDING:

The project described was supported by Award Number U01AI068636 from the National Institute of Allergy and Infectious Diseases and supported by National Institute of Mental Health (NIMH), National Institute of Dental and Craniofacial Research (NIDCR). The content is solely the responsibility of the authors and does not necessarily represent the official views of the National Institute of Allergy and Infectious Diseases or the National Institutes of Health.

The ACTG sites and site investigators were also supported NIH grants AI069513, AI34835, AI069432, AI069423, AI069477, AI069501, AI069474, AI069428, AI69467, AI069415, Al32782, AI27661, AI25859, AI28568, AI30914, AI069495, AI069471, AI069532, AI069452, AI069450, AI069556, AI069484, AI069472, AI34853, AI069465, AI069511, AI38844, AI069424, AI069434, AI46370, AI68634, AI069502, AI069419, AI69494, AI69447, AI69439, AI69503, AI69470, AI69503, RR024975, AI077505, and TR000445.

***The AIDS Linked to the IntraVenous Experience (ALIVE) Cohort***

DESCRIPTION:

The *AIDS Linked to the IntraVenous Experience* (ALIVE) study is a natural history study following the incidence and progression of HIV infection among intravenous drug users (IDUs) in Baltimore, MD.

MEMBERS:

Gregory D. Kirk, Shruti H. Mehta, Kenrad Nelson, Steffanie Strathdee, David Vlahov

FUNDING:

National Institutes of Health grants: R01-DA-04334 and R01-DA-12568

***The Amsterdam Cohort Studies on HIV infection and AIDS (ACS)***

DESCRIPTION:

The Amsterdam Cohort Studies (ACS) on HIV infection and AIDS were started shortly after the first cases of AIDS were diagnosed in the Netherlands. Since October 1984, men who have sex with men (MSM) have been enrolled in a prospective cohort study. A second cohort involving drug users (DU) was initiated in 1985.

MEMBERS:

Neeltje A. Kootstra, Daniëlle van Manen, Brigitte Boeser-Nunnink, Angélique B. van ’t Wout, Hanneke Schuitemaker

FUNDING:

The Amsterdam Cohort Studies on HIV infection and AIDS, a collaboration between the Public Health Service of Amsterdam, the Academic Medical Center of the University of Amsterdam, the Sanquin Blood Supply Foundation, the University Medical Center Utrecht, and the Jan van Goyen Medical Center, are part of the Netherlands HIV Monitoring Foundation and financially supported by the Center for Infectious Disease Control of the Netherlands National Institute for Public Health and the Environment. We acknowledge funding from the Netherlands Organization for Scientific Research (TOP, registration number 9120.6046)

***The ANRS CO18***

DESCRIPTION:

The ANRS CO18 cohort prospectively enrolls HIV controllers, defined as patients infected by HIV-1 for ≥ 5 years who have never received antiretroviral treatment and whose last 5 consecutive plasma HIV RNA values are < 400 copies/mL.

MEMBERS:

Olivier Lambotte, Laurence Meyer, Faroudy Boufassa, Christine Rouzioux, Asier Saez-Cirion, Gianfranco Pancino, Alain Venet, Camille Lecuroux, Ioannis Theodorou, Lisa Chakrabarti

FUNDING:

ANRS, Sidaction, University Paris Sud

***The ANRS PRIMO Cohort***

DESCRIPTION:

The ANRS CO06 PRIMO is an ongoing multicenter cohort that enrolls patients with demonstrated primary HIV-1 infection in France since November 1996.

MEMBERS:

Région Sud–Est : André-Pierre BLANC, Thierry ALLEGRE, Claudine LAFON, Isabelle ROUBAUD, Jean-Louis TOURAINE, Jean-Michel LIVROZET, François JEANBLANC, Pierre CHIARELLO, Djamila MAKHLOUFI, Dominique PEYRAMOND, Christian TREPO, Patrick MIAILHES, Joseph KOFFI, Thomas BAUDRY, Sylvie PAILHES, Valérie THOIRAIN, Corinne BROCHIER, Alain LAFEUILLADE, Antoine CHERET, Julie ALLEMAND, Thomas HULEUX, Gilles HITTINGER, Alain RIEU, Gisèle PHILIP, Assi ASSI, Edith DANIELLI, Véronique LAMBRY, Pierre DELLAMONICA, Jacques DURANT, Eric CUA, Anne LEPLATOIS, Pascal PUBLIESE, Alissa NAQVI, Véronique MONDAIN –MITON, Matteo VASSALLO, Marie-Ange SERINI, Jean-Gabriel FUZIBET, Jill-Patrice CASSUTO, Michèle QUARANTA, Claudine SOHN, Eric ROSENTHAL, Isabelle POIZOT MARTIN, Marie-Pierre DROGOUL, Jean-Albert GASTAUT, Geneviève FABRE, Caroline DEBREUX, Andréas STEIN, Isabelle RAVAUX, Hervé GALLAIS, Annette VALON, Gilles PICHANCOURT, Denis QUINSAT, Serge TEMPESTA, Sylvie ANDREO, Patrick PHILIBERT, Christine CHAPUS, Robert RIOU, Hélène CHAMPAGNE

Région Sud-Ouest : Philippe MORLAT, Fabrice BONNET, Isabelle LOUIS, Caroline ASLAN, Denis MALVY, Denis LACOSTE, Noëlle BERNARD, François PACCALIN, Mojgan BONAREK, Marie-Anne VANDENHENDE, Jean DELAUNE, Sabrina CALTADO, Jean-Marie RAGNAUD, Michel DUPON, Hervé DUTRONC, Frédéric DAUCHY, Charles CAZENAVE, Didier NEAU, Laetitia LACAZE-BUZY, Thierry PISTONE, Elodie BLANCHARD, Audrey MERLET, Jean-Luc PELLEGRIN, Isabelle RAYMOND, Jean-François VIALLARD, Estibaliz LAZARO, Carine GREIB, Gaetane WIRTH, Severin CHAIGNE DE LALANDE, Jacques REYNES, Vincent BAILLAT, Corinne MERLE de BOEVER, Vincent LEMOING, Nadine ATOUI, Alain MAKINSON, Jean Marc JACQUET, Christina PSOMAS, Antoine VILLADERO, Christine TRAMONI, Michèle VIDAL, Bruno MARCHOU, Martine OBADIA, Marie CHAUVEAU, Florence BALZARIN, Francis SAINT-DIZIER, Daniel GARIPUY, Roland VIRABEN, François PREVOTEAU du CLARY, Hugues AUMAITRE, Mathieu SAADA, Marie MEDUS, Orélia EDEN, Ségolène NEUVILLE, Milagros FERREYRA, Martine MALET

Antilles : André CABIE, Sylvie ABEL, Sandrine PIERRE-FRANCOIS, Guillaume HURTREL, Patrick HOCHEDEZ, Guillaume AVENIN, Nicolas VIGNIER, Bernard LIAUTAUD, Christelle BARINGTHON, Véronique BEAUJOLAIS, Gilles BEAUCAIRE, Isabelle LAMAURY

Région Est : Bernard DRENOU, Geneviève BECK-WIRTH, Catherine MICHEL, Jean-Michel PETER, Charles BECK, Jean-Michel HALNA, Meryem BENOMAR, Bruno HOEN, Christine DROBACHEFF-THIEBAUT, Jean-François FAUCHER, Adeline FOLTZER, François PARCELIER, Catherine BOURDEAUX, Jean-Marie ESTAVOYER, Lionel PIROTH, Marielle BUISSON, Sandrine TREUVELOT, Thierry MAY, Laurence BOYER, Simone WASSOUMBOU, Bruno AUDHUY, Masha MOHSENI ZADEH, Martin MARTINOT, Nathalie PLAISANCE, Anaïs MOTHES, Anne PACHART, Elisabeth SALLE, Benoît MARTHA, Marguerite FLORENCE, David REY, Maria PARTISANI, Christine CHENEAU, Michèle PRIESTER, Claudine BERNARD-HENRY, Erick de MAUTORT, Patricia FISCHER, Roland JAUSSAUD, Christine ROUGER, Jean-Luc BERGER

Région Nord : Eric SENNEVILLE, Antoine CHERET, Thomas HULEUX, Faïza AJANA, Isabelle ALCARAZ, Christophe ALLIENNE, Véronique BACLET, Xavier de la TRIBONNIERE, Hugues MELLIEZ, Agnès MEYBECK, Bertrand RIFF, Michel VALETTE, Nathalie VIGET, Emmanuelle AISSI, Yves DOMART, Dominique MERRIEN, Jean-Luc SCHMIT, Youssef EL SAMAD, Nathalie DECAUX, Georges DIAB, Jean GRIHON

Région Ouest: François RAFFI, Bénédicte BONNET, Clotilde ALLAVENA, Jean-Philippe TALARMIN, Olivier MOUNOURY, Véronique RELIQUET, Eric BILLAUD, Cécile BRUNET, Sabelline BOUCHEZ, Nicolas FEUILLEBOIS, David BOUTOILLE, Hervé HUE, Delphine BROSSEAU, Myriam SICOT, Christian MICHELET, Faouzi SOUALA, Pierre TATTEVIN, Cédric ARVIEUX, Matthieu REVEST, Hélène LEROY, Jean-Marc CHAPPLAIN, Matthieu DUPONT, Fabien FILY, Maja RASJTAJACK, Louis BERNARD, Frédéric BASTIDES, Pascale NAU, Vanessa LAPLANTINE, Renaud VERDON, Arnaud de la BLANCHARDIERE, Vincent NOYON, Anne MARTIN, Mathilde SIX, Philippe FERET, Claude BEUSCART, Corinne DANIEL, Sylvie Le MOAL, Elizabeth DUHAMEL, Anne GATEL, Elisabeth PAPE, Eric PICHARD, Pascale FIALAIRE, Jean Marie CHENNEBAULT, Jocelyne LOISON, Valérie RABIER, Pierre ABGUEGUEN, Sami REHAIEM, Philippe MOREAU, Odile VAILLANT, Mathilde NIAULT, Yves POINSIGNON, Anabèle DOS SANTOS, Virginie MOUTON- RIOUX, Bernadette Le MABEC, Dominique HOULBERT, Philippe PERRE, Sophie LEANTEZ-NAINVILLE, Jean-Luc ESNAULT, Thomas GUIMARD, Isabelle SUAUD, Jean-Jacques GIRARD, Véronique SIMONET, Loïk GEFFRAY, François CARON, Yasmine DEBAB

Région Centre : Jean BEYTOUT, Christine JACOMET, Florence GOURDON, Hacène TAS, Pierre WEINBRECK, Claire GENET, Grégoire LAMBERT de CURSAY, Bruno ABRAHAM, Claire PERINO, Pauline PINET, Alain REGNIER, Odile ANTONIOTTI

Région Parisienne : Jean-François DELFRAISSY, Cécile GOUJARD, Jade GHOSN, Yann QUERTAINMONT, Martin DURACINSKY, Olivier SEGERAL, Arnaud BLANC, Delphine PERETTI, Valérie SEGUIN, Erwan FOURN, Marie-Thérèse RANNOU, Marie Josée DULUCQ, Philippe BROET, Cyril DALMASSO, Daniel VITTECOQ, Lelia ESCAUT, Claudine BOLLIOT, Yves LEVY, Anne-Sophie LASCAUX, Jean-Daniel LELIEVRE, Stéphanie DOMINGUEZ, Corinne JUNG, Cécile DUMONT, François BOUE, Renato FIOR, Véronique CHAMBRIN, Agnès LEVY, Valérie MARTINEZ, Christian PERRONNE, Pierre de TRUCHIS, Huguette BERTHE, Pierre CHEVOJON, Alain DEVIDAS, Isabelle TURPAULT, Fabrice CHAIX, Isabelle TURPAULT, Olivier BOUCHAUD, François ROUGES, Sophie ABGRALL, Régine BARRUET, Frédéric MECHAI, Patricia HONORE, Elisabeth ROUVEIX, Evelyne REIMANN, Alix GREDER BELAN, Audrey THERBY, Claire GODIN COLLET, Maria RUQUET, Isabelle MAHE, Anne-Marie SIMONPOLI, Emmanuel MORTIER, Martine BLOCH, Véronique MANCERON, Isabelle CAHITTE, Ai-feng ZENG, Bénédicte MONTOYA, David ZUCMAN, Catherine MAJERHOLC, Dominique BORNAREL, Bruno FANTIN, Agnès ULUDAG, Justine GELLEN-DAUTREMER, Agnès. LEFORT, Ali.TADLAOUI, Michel RUEL, Vincent DANELUZZI, Juliette GERBE, Olivier FEIN, Jérôme STIRNEMANN, Virginie PRENDKI, Sylvie TASSI, Mélissa COUPARD, Olivier PATEY, Jonas BANTSIMBA, Sophie DELLION, Pauline CARAUX PAZ, Benoit CAZENAVE, Laurent RICHIER, Isabelle de LACROIX SZMANIA, Valérie GARRAIT, Brigitte ELHARRAR, Laurent RICHIER, Annie LEPRETRE, Marie-Agnès KOZA, Laurent SUTTON, Philippe GENET, Juliette GERBE, Françoise GRANIER, Véronique PERRONE, Jean-Luc BOUSSARD, Patricia CHARDON, Eric FROGUEL, Philippe SIMON, Sylvie TASSI, Jean-Michel MOLINA, Samuel FERRET, Matthieu LAFAURIE, Nathalie COLIN de VERDIERE, Diane PONSCARME, Nathalie de CASTRO, Alexandre ASLAN, Willy ROZENBAUM, Jerôme GOGUEL, Suna BALKAN, Sébastien GALLIEN, François CLAVEL, Caroline GATEY, Valérie GARRAIT, Bénédicte LOZE, Caroline LASCOUX-COMBE, Olivier TAULERA, Claire PINTADO, Jeannine DELGADO, Daniel SERENI, Patrice MOREL, Julie TIMSIT, Françoise LAMBERT, Eric OKSENHENDLER, Laurence GERARD, Pierre-Marie GIRARD, Odile PICARD, Jürgen TREDUP, Diane BOLLENS, Nadia VALIN, Pauline CAMPA, Nelly DESPLANQUE, Yazdan YAZDANPANAH, Patrick YENI, Elisabeth BOUVET, Isabelle FOURNIER, Bao PHUNG, Bilguissa DIALLO, Frédérique GUIROY, Nadia EL ALAMI TALBI, Golriz PAHALAVAN, Françoise LOUNI, Catherine LEPORT, Corinne JADAND, Ugo COLASANTE, Jean-Jacques LAURICHESSE, Christine JESTIN, Gilles PIALOUX, Laurence SLAMA, Thomas LYAVANC, Valérie BERREBI, Firouze BANI SADR, Jean-François BERGMAN, Myriam DIEMER, Aurélie DUREL, Agathe RAMI, Maguy PARRINELLO, Loïc GUILLEVIN, Dominique SALMON, Tassadit TAHI, Catherine CHAKVETADZE, Linda BELARBI, Odile LAUNAY, Benjamin SILBERMANN, Marie-Pierre PIETRI, François BRICAIRE, Christine KATLAMA, Marc-Antoine VALANTIN, Roland TUBIANA, Fabienne CABY, Luminita SCHNEIDER, Sophie SEANG, Hind STITOU, Saadia BEN ABDALLAH, Serge HERSON, Anne SIMON, Manuela BONMARCHAND, Naouel AMIRAT, François PICHON, Jean-Luc VOURCH, Michèle PAUCHARD, Nadia ADEB, Laurence WEISS, Martin BUISSON, Maria MANEA, Jean-Paul VIARD, Jacques GILQUIN, Alain SOBEL, Blanka HADACEK, Nugyen THU-HUYN, Olivier ZAK-DIT-ZBAR, Agnès CROS, Paul Henri CONSIGNY, Claudine DUVIVIER, Fatima TOUAM, Loïc BODARD, Isabelle SAURET, Sylvie GIBERT, Anne MEUDEC, Gérard HUCHON, Alejandra COMPAGNUCCI, Christian MIODOVSKI

FUNDING:

ANRS

**The Center for Cancer Research, National Cancer Institute**

DESCRIPTION:

The Center for Cancer Research (CCR) is home to more than 250 scientists and clinicians working in intramural research at NCI. CCR's investigators are basic, clinical, and translational scientists who work together to advance knowledge of cancer and AIDS and to develop new therapies against these diseases.

MEMBERS:

Ping An, Sher Hendrickson, Randall Johnson, Bailey Kessing, James Lautenberger, Carl McIntosh, George Nelson, Stephen O’Brien, Efe Szegin, Jennifer Troyer, and Cheryl Winkler

FUNDING:

This research was supported by the Intramural Research Program of NIH, Frederick National Laboratory, Center for Cancer Research, National Cancer Institute and also funded in whole or in part with Federal funds from the Frederick National Laboratory for Cancer Research, National Institutes of Health, under contract HHSN261200800001E.

***The Center for HIV/AIDS Vaccine Immunology (CHAVI)***

DESCRIPTION:

The Center for HIV/AIDS Vaccine Immunology (CHAVI) is a consortium of universities and academic medical centers that was established by the National Institute of Allergy and Infectious Diseases (NIAID) from 2005-2012. CHAVI's goal was to solve the major problems in HIV vaccine development and design.

MEMBERS:

CHAVI is led by Barton Haynes (Duke University, Durham, NC, USA). Its Host Genetics Core is led by David Goldstein (Duke University, Durham, NC, USA).

FUNDING:

Funding for research by CHAVI was provided by NIH NIAID grant AI067854

***The Danish HIV Cohort Study***

DESCRIPTION:

The Danish HIV Cohort Study (DHCS) includes all HIV-infected individuals, aged 16 years or older at time of HIV diagnosis, seen in one of the eight Danish HIV centres after 31 December 1994. The aims of DHCS are (i) to create a population-based nationwide database of HIV patients for scientific projects, (ii) to monitor the spread and demographics of the HIV epidemic in Denmark, (iii) to monitor and compare the effects of treatment strategies in Danish HIV treatment centres and (iv) to monitor the effect of HAART on a national level.

MEMBERS:

Neils Obel, Jan Gerstoft, Gitte Kronborg, Court Pedersen

FUNDING:

The Danish HIV Cohort Study is funded mainly by Copenhagen University, University of Southern Denmark and the Danish AIDS Foundation

***The Genetic and Immunological Studies of European and African HIV-1+ Long Term Non-Progressors (GISHEAL) Study***

DESCRIPTION:

GISHEAL is a collaborative European consortium focusing on long-term nonprogressors (LTNP), with particular regard to the host genetic background and gene expression profiling and to the adaptive and innate immunological responses to the infection.

MEMBERS:

Guido Poli (Coordinator); steering committee: Brigitte Autran (Vice-Coordinator),

Dominique Costagliola, Patrice Debré, Massimo Galli, Frances Gotch, Pontiano Kaleebu, Federico Martini, Agostino Riva, Ioannis Theodorou, Elisa Vicenzi,

FUNDING:

6^th^ Framework Programme, EU

***The GRIV Cohort***

DESCRIPTION:

The GRIV cohort, started in 1995 in France, includes both fast and slow/non-progressors. It aims at understanding basic features of the yet unknown HIV pathogenesis mechanisms.

MEMBERS:

Cédric Coulonges, Olivier Delaneau, Lieng Taing, Pierre Rucart, Sigrid Le Clerc, Sophie Limou, Jean-François Zagury.

FUNDING:

The GRIV cohort genome-wide analysis was funded by Agence Nationale de Recherches sur le SIda et les Hépatites Virales (ANRS), Conservatoire National des Arts et Métiers (Cnam), and Peptinov. ANRS funded in particular the P3S Genomic platform (hospital La Pitié Salpétrière) that was used for genotyping the cohort.

Olivier Delaneau was funded by post-doctoral grants from ANRS and Peptinov.

Pierre Rucart has a post-doctoral fellowship from ANRS

***The Hemophilia Growth and Development Study (HGDS)***

DESCRIPTION:

The HGDS is a multicenter study of hemophilia and its complications that was established in 1988. Data were prospectively collected in 14 U.S. hemophilia treatment centers through 1996/97. A total of 333 children and adolescents were enrolled. The HGDS has investigated the effects of hemophilia and HIV on physical growth and maturation; immunological, neurological, and neuropsychological functioning; and the pathophysiology of HIV and hepatitis C.

MEMBERS:

Eric Daar, Sharyne Donfield, Edward Gomperts, Margaret Hilgartner, W. Keith Hoots, Henry Lynn, Anne Willoughby, Cheryl Winkler

FUNDING:

National Institutes of Health, National Institute of Child Health and Human Development, R01-HD-41224

***The Hospital Clinic-IDIBAPS Acute/Recent HIV-1 Infection cohort***

DESCRIPTION:

The Hospital Clinic PHI Cohort was created in 1997 and it has a biobank of biological and tissue samples since 2004. It focuses on studies of transmitted drug resistance and in clinical trials with new antiretroviral drugs and/or immune modulators trying to change the natural history of HIV-infection.

MEMBERS:

J. M. Miro (Chair), C. Manzardo, F. Agüero, J. A. Arnaiz, T. Pumarola, M. Plana, M. Tuset, M. C. Ligero, VM Sanchez, T. Gallart, and J. M. Gatell.

FUNDING:

The Hospital Clinic-IDIBAPS Acute/Recent HIV-1 Infection cohort in Barcelona, Spain (JMM) has been in part funded by the ‘Red Temática Cooperativa de Grupos de Investigación en Sida del Fondo de Investigación Sanitaria (FIS)”, and the grant ISCIII-RETIC RD06/006 from the “Instituto de Salud Carlos III”, Madrid Spain.

***The Icona Foundation Study***

DESCRIPTION:

The Italian cohort of patients naive from antiretrovirals (ICONA) was set up in April 1997. ICONA is a multicentre, observational study. By September 2006 a total of 6,205 naive patients had been enrolled. The main focus of ICONA is the outcome of antiretroviral therapy.

MEMBERS:

GOVERNING BODY: M. Moroni (Chair), G Angarano, A Antinori, F Castelli, R Cauda, A d’Arminio Monforte, G Di Perri, M Galli, R Iardino, G Ippolito, A Lazzarin, CF Perno, O Armignacco, PL Viale, F Von Schlosser.

SCIENTIFIC SECRETARY:

A d’Arminio Monforte

STEERING COMMITTEE: A Ammassari, M Andreoni, A Antinori, C Balotta, P Bonfanti, S Bonora, M Borderi, MR Capobianchi, A Castagna, F Ceccherini-Silberstein, P Cinque, A Cozzi-Lepri, A d’Arminio Monforte, A De Luca, M Gargiulo, C Gervasoni, E Girardi, A Gori, G Guaraldi, M Lichtner, S Lo Caputo, G Madeddu, F Maggiolo, G Marchetti, S Marcotullio, L Monno, R Murri, C Mussini, M Puoti, C Torti

STATISTICAL AND MONITORING TEAM

A Cozzi-Lepri, P Cicconi, I Fanti, T Formenti, L Galli, P Lorenzini

PARTICIPATING PHYSICIANS AND CENTERS: Italy A. Giacometti, A Costantini, A. Riva (Ancona); G. Angarano, L Monno, C Carrisa, (Bari); F. Maggiolo, G Lazzari (Bergamo); PL. Viale, M Borderi, G. Verucchi (Bologna); F Castelli, C. Torti, C. Minardi, (Brescia); T. Quirino, C Abeli (Busto Arsizio); P.E. Manconi, P. Piano (Cagliari); J Vecchiet, K Falasca (Chieti); L. Sighinolfi, D. Segala (Ferrara); F. Mazzotta, S. Lo Caputo (Firenze); G. Cassola, G Viscoli, A. Alessandrini, R. Piscopo, G Mazzarello (Genova); C. Mastroianni, V. Belvisi (Latina); P. Bonfanti, I. Caramma (Lecco); A. Chiodera, P. Castelli (Macerata); M Galli, A. Lazzarin, G. Rizzardini, M. Puoti, A. d’Arminio Monforte, AL Ridolfo, R Piolini, A Castagna, S Salpietro, A Galli, A Bigoloni, V Spagnuolo, L Carenzi, P Zucchi, M.C. Moioli, R Rossotti, P Cicconi, T Formenti (Milano); C. Mussini, L Bisio (Modena); A Gori, G Lapadula (Monza), N. Abrescia, A. Chirianni, MG Guida, M Gargiulo (Napoli); F Baldelli, B Belfiori (Perugia); G. Parruti, T Ursini (Pescara); G. Magnani, M.A. Ursitti (Reggio Emilia); R. Cauda, M Andreoni, A. Antinori, V Tozzi, V. Vullo, A. De Luca, A. d’Avino, M. Zaccarelli, L Gallo, E. Nicastro, R. Acinapura, M Capozzi, R Libertone, M. Lichtner, G Tebano, (Roma); M.S. Mura, G Madeddu (Sassari); P. Caramello, G. Di Perri, G.C. Orofino, M Sciandra (Torino); G. Pellizzer, V. Manfrin (Vicenza).

***The International HIV Controllers Study***

DESCRIPTION:

The International HIV Controllers Study is a collaborative effort among scientists, healthcare professionals and the community to study HIV infected people who have been able to maintain low viral loads without the use of medications.

MEMBERS:

HIV controllers recruitment and sample management: Florencia Pereyra, Alicja Piechocka-Trocha, Emily Cutrell, Rachel Rosenberg, Kristin L. Moss, Ildiko Toth, Brian Block, Brett Baker, Alissa Rothchild, Jeffrey Lian, Jacqueline Proudfoot, Marylyn M. Addo, Bruce D. Walker

HIV controllers referral team: Brian Agan, Shanu Agarwal, Richard L. Ahern, Brady L. Allen, Sherly Altidor, Eric L. Altschuler, Sujata Ambardar, Kathryn Anastos, Val Anderson, Ushan Andrady, Diana Antoniskis, David Bangsberg, Daniel Barbaro, William Barrie, J. Bartczak, Simon Barton, Patricia Basden, Nesli Basgoz, Nicholaos C. Bellos, Judith Berger, Nicole F. Bernard, Annette M. Bernard, Stanley J. Bodner, Robert K. Bolan, Emilie T. Boudreaux, James F. Braun, Jon E. Brndjar, J. Brown, Sheldon T. Brown, Jedidiah Burack, Larry M. Bush, Virginia Cafaro, John Campbell, Robert H. Carlson, J. Kevin Carmichael, Kathleen K. Casey, Chris Cavacuiti, Gregory Celestin, Steven T. Chambers, Nancy Chez, Lisa M. Chirch, Paul J. Cimoch, Daniel Cohen, Lillian E. Cohn, Brian Conway, David A. Cooper, Brian Cornelson, David T. Cox, Michael V. Cristofano, George Cuchural Jr., Julie L. Czartoski, Joseph M. Dahman, Jennifer S. Daly, Benjamin T. Davis, Kristine Davis, Sheila M. Davod, Steven G. Deeks, Edwin DeJesus, Craig A. Dietz, Eleanor Dunham, Michael E. Dunn, Todd B. Ellerin, Joseph J. Eron, John J.W. Fangman, Helen Ferlazzo, Sarah Fidler, Anita Fleenor-Ford, Renee Frankel, Kenneth A. Freedberg, Neel K. French, Jonathan D. Fuchs, Jon D. Fuller, Jonna Gaberman, Joel E. Gallant, Rajesh T. Gandhi, Efrain Garcia, Donald Garmon, Joseph C. Gathe Jr, Cyril R. Gaultier, Wondwoosen Gebre, Frank D. Gilman, Ian Gilson, Paul A. Goepfert, Michael S. Gottlieb, Claudia Goulston, Richard K. Groger, T. Douglas Gurley, Stuart Haber, Robin Hardwicke, W. David Hardy, P. Richard Harrigan, Trevor N. Hawkins, Sonya Heath, Frederick M. Hecht, W. Keith Henry, Melissa Hladek, Robert P. Hoffman, James M. Horton, Ricky K. Hsu, Gregory D. Huhn, Peter Hunt, Mark L. Illeman, Hans Jaeger, Robert M. Jellinger, Mina John, Jennifer A. Johnson, Kristin L. Johnson, Heather Johnson, Kay Johnson, Jennifer Joly, Wilbert C. Jordan, Carol A. Kauffman, Homayoon Khanlou, Arthur Y. Kim, David D. Kim, Clifford A. Kinder, Laura Kogelman, Erna Milunka Kojic, P. Todd Korthuis, Wayne Kurisu, Douglas S. Kwon, Melissa LaMar, Harry Lampiris, Michael M. Lederman, David M. Lee, Marah J. Lee, Edward T.Y. Lee, Janice Lemoine, Jay A. Levy, Josep M. Llibre, Michael A. Liguori, Susan J. Little, Anne Y. Liu, Alvaro J. Lopez, Mono R. Loutfy, Dawn Loy, Debbie Y. Mohammed, Alan Man, Michael K. Mansour, Vincent C. Marconi, Martin Markowitz, Jeffrey N. Martin, Harold L. Martin Jr., Kenneth Hugh Mayer, M. Juliana McElrath, Theresa A. McGhee, Barbara H. McGovern, Katherine McGowan, Dawn McIntyre, Gavin X. McLeod, Prema Menezes, Greg Mesa, Craig E. Metroka, Dirk Meyer-Olson, Andy O. Miller, Kate Montgomery, Karam C. Mounzer, Iris Nagin, Ronald G. Nahass, Craig Nielsen, David L. Norene, David H. O'Connor, Jason Okulicz, Edward C. Oldfield III, Susan A. Olender, Mario Ostrowski, William F. Owen Jr., Jeffrey Parsonnet, Andrew M. Pavlatos, Aaron M. Perlmutter, Jonathan M. Pincus, Leandro Pisani, Lawrence Jay Price, Laurie Proia, Richard C. Prokesch, Heather Calderon Pujet, Moti Ramgopal, Michael Rausch, J. Ravishankar, Frank S. Rhame, Constance Shamuyarira Richards, Douglas D. Richman, Gregory K. Robbins, Berta Rodes, Milagros Rodriguez, Richard C. Rose III, Eric S. Rosenberg, Daniel Rosenthal, Polly E. Ross, David S. Rubin, Elease Rumbaugh, Luis Saenz, Michelle R. Salvaggio, William C. Sanchez, Veeraf M. Sanjana, Steven Santiago, Wolfgang Schmidt, Hanneke Schuitemaker, Philip M. Sestak, Peter Shalit, William Shay, Vivian N. Shirvani, Vanessa I. Silebi, James M. Sizemore Jr., Paul R. Skolnik, Marcia Sokol-Anderson, James M. Sosman, Paul Stabile, Jack T. Stapleton, Francine Stein, Hans-Jurgen Stellbrink, F. Lisa Sterman, Valerie E. Stone, David R. Stone, Giuseppe Tambussi, Randy A. Taplitz, Ellen M. Tedaldi, Amalio Telenti, Richard Torres, Lorraine Tosiello, Cecile Tremblay, Marc A. Tribble, Phuong D. Trinh, Anthony Vaccaro, Emilia Valadas, Thanes J. Vanig, Isabel Vecino, Wenoah Veikley, Barbara H. Wade, Charles Walworth, Chingchai Wanidworanun, Douglas J. Ward, Robert D. Weber, Duncan Webster, Steve Weis, David A. Wheeler, David J. White, Ed Wilkins, Alan Winston, Clifford G. Wlodaver, Angelique van 't Wout, David P. Wright, Otto O. Yang, David L. Yurdin, Brandon W. Zabukovic, Kimon C. Zachary, Beth Zeeman, Meng Zhao

FUNDING:

The International HIV Controllers Study was made possible through a generous donation from the Mark and Lisa Schwartz Foundation and a subsequent award from the Collaboration for AIDS Vaccine Discovery (CAVD) of the Bill and Melinda Gates Foundation. This work was also supported in part by the Harvard University Center for AIDS Research (P-30- AI060354), UCSF CFAR (P-30 AI27763), UCSF CTSI (UL1 RR024131), CNICS (R24 AI067039), NIH grants AI28568, AI030914 (B.D.W.); AI087145, K24AI069994 (S.G.D.)

***The IrsiCaixa Foundation Acute/Recent HIV-1 Infection Cohort***

DESCRIPTION:

The IrsiCaixa Institute for AIDS Research aims at improving understanding of the disease, its prevention and treatments of HIV and AIDS with the ultimate goal of eradicating the epidemic.

MEMBERS:

Judith Dalmau, Javier Martinez-Picado, Bonaventura Clotet, Eulàlia Grau, Rafaela Ayen, Jordi Puig, Cristina Miranda, José Ramón Santos, Ingrid Martínez, Pere Domingo, David Dalmau, José Moltó, Robert Muga, Guilem Sirera, Maria del Mar Gutiérrez, Maria Gràcia Mateo

FUNDING:

This study was supported in part by Fondo de Investigaciones Sanitarias (PI061259), Red de Investigación en Sida (RD06/0006) and HIVACAT (Projecte de recerca de la vacuna de la SIDA)

***The Modena Cohort***

DESCRIPTION:

The Modena HIV Surveillance Cohort collects data concerning new cases of HIV infected adults since 1985.

MEMBERS:

Andrea Cossarizza

FUNDING:

Istituto Superiore di Sanità, Rome (Italy), grant: 40H71

***The Multicenter AIDS Cohort Study (MACS)***

DESCRIPTION:

The Multicenter AIDS Cohort Study (MACS) is an ongoing prospective study of the natural and treated histories of HIV-1 infection in homosexual and bisexual men conducted by sites located in Baltimore, Chicago, Pittsburgh and Los Angeles. A total of 6,972 men have been enrolled.

MEMBERS:

**Baltimore**: The Johns Hopkins University Bloomberg School of Public Health: Joseph B. Margolick (Principal Investigator), Barbara Crain, Adrian Dobs, Homayoon Farzadegan, Joel Gallant, Lisette Johnson, Shenghan Lai, Ned Sacktor, Ola Selnes, James Shepherd, Chloe Thio.

**Chicago**: Howard Brown Health Center, Feinberg School of Medicine, Northwestern University, and Cook County Bureau of Health Services: John P. Phair and Steven Wolinsky (Multiple Principal Investigators), Sheila Badri, Bruce Cohen, Craig Conover, Maurice O'Gorman, David Ostrow, Frank Palella.

**Los** **Angeles**: University of California, UCLA Schools of Public Health and Medicine: Roger Detels (Principal Investigator), Barbara R. Visscher (Co-Principal Investigator), Aaron Aronow, Robert Bolan, Elizabeth Breen, Anthony Butch, Thomas Coates, Rita Effros, John Fahey, Beth Jamieson, Otoniel Martínez-Maza, Eric N. Miller, John Oishi, Paul Satz, Harry Vinters, Dorothy Wiley, Mallory Witt, Otto Yang, Stephen Young, Zuo Feng Zhang.

**Pittsburgh**: University of Pittsburgh, Graduate School of Public Health: Charles R. Rinaldo (Principal Investigator), Lawrence Kingsley (Co-Principal Investigator), James T. Becker, Robert W. Evans, John Mellors, Sharon Riddler, Anthony Silvestre.

**Data Coordinating Center**: The Johns Hopkins University Bloomberg School of Public Health: Lisa P. Jacobson (Principal Investigator), Alvaro Muñoz (Co-Principal Investigator), Keri Althoff, Christopher Cox, Gypsyamber D'Souza, Stephen J. Gange, Elizabeth Golub, Janet Schollenberger, Eric C. Seaberg, Sol Su.

FUNDING:

NIH: National Institute of Allergy and Infectious Diseases: Robin E. Huebner; National Cancer Institute: Geraldina Dominguez; National Heart, Lung and Blood Institute: Cheryl McDonald. UO1-AI-35042, 5-M01-RR-00052 (GCRC), UO1-AI-35043, UO1-AI-37984, UO1-AI-35039, UO1-AI-35040, UO1-AI-37613, and UO1-AI-35041. This work was also supported in part by NIH grants R37 AI47734 (J.I.M.); T32 AI07140 (J.T.H.), and the University of Washington Center for AIDS Research, an NIH funded program (P30 AI027757), which is supported by the following NIH Institutes and Centers (NIAID, NCI, NIMH, NIDA, NICHD, NHLBI, NIA).

***The Multicenter Hemophilia Cohort Studies (MHCS)***

DESCRIPTION:

The first Multicenter Hemophilia Cohort Study (MHCS-I) evaluated and prospectively followed patients with hemophilia or a related coagulation disorder. Initiated in 1982, this study particularly sought to understand the cause and natural history of HIV infection and AIDS in this population, which was at high risk for development of AIDS.

MEMBER:

James J. Goedert.

FUNDING:

Intramural Research Program, National Cancer Institute, National Institutes of Health

***The Pumwani Sex Workers Cohort***

DESCRIPTION:

The Nairobi (Pumwani) commercial sex worker cohort was established in 1985. Despite repeated exposures to HIV-1, a number of women in this cohort have remained HIV-1 uninfected for long periods of time and have been epidemiologically defined as HIV resistant.

MEMBERS:

Francis A. Plummer, Terry Blake Ball, Keith Fowke, Joshua Kimani, Larry Gelmon, Ma Luo, Elizabeth Ngugi

FUNDING:

The Pumwani Sex Workers Cohort has been supported by various grants from Canadian Institute of Health Research, Canadian International Development Agency, National Institute of Health (R01 AI56980), USA and Bill and Melinda Gates Foundation since 1985. This work was supported by a grant from the Bill and Melinda Gates Foundation and the Canadian Institutes of Health Research (HOP-43135) through the Grand Challenges in Global Health Initiative.

***The San Francisco City Clinic Cohort (SFCCC)***

DESCRIPTION:

The San Francisco City Clinic Cohort (SFCCC) consists of approximately 6700 homosexual men recruited between 1978 and 1980 from a clinic for sexually transmitted diseases.

MEMBERS:

Susan Buchbinder

FUNDING:

This was supported by cooperative agreement (No U62/CCU900523) from the

Centers for Disease Control, Atlanta, Georgia

***The GPC Cohort / Sanger Institute in Oxford, UK, and in Uganda***

DESCRIPTION:

The General Population Cohort (GPC) was set up in 1989 to examine trends in HIV prevalence and incidence, and their determinants in rural south-western Uganda. Recently, the research questions have included the epidemiology and genetics of communicable and non-communicable diseases (NCDs) to address the limited data on the burden and risk factors for NCDs in sub-Saharan Africa.

MEMBER:

Manj S Sandhu

***The Swiss HIV Cohort Study (SHCS)***

DESCRIPTION:

The Swiss HIV Cohort Study (SHCS) is an ongoing multi-center research project dealing with HIV infected adults aged 16 years or older. Since it was established in 1988, the SHCS recruited and followed more than 17’000 patients in seven centers.

MEMBERS:

Aubert V, Barth J, Battegay M,  Bernasconi E, Böni J, Bucher HC,  Burton-Jeangros C, Calmy A, Cavassini M, Egger M, Elzi L, Fehr J, Fellay J, Francioli P, Furrer H (Chairman of the Clinical and Laboratory Committee), Fux CA, Gorgievski M, Günthard H (President of the SHCS), Haerry D (deputy of "Positive Council"), Hasse B, Hirsch HH, Hirschel B, Hösli I, Kahlert C, Kaiser L, Keiser O, Kind C, Klimkait T, Kovari H, Ledergerber B, Martinetti G, Martinez de Tejada B, Metzner K, Müller N, Nadal D, Pantaleo G, Rauch A (Chairman of the Scientific Board),  Regenass S, Rickenbach M (Head of Data Center), Rudin C (Chairman of the Mother & Child Substudy), Schmid P, Schultze D, Schöni-Affolter F,  Schüpbach J, Speck R,  Taffé P, Tarr P, Telenti A, Trkola A,  Vernazza P, Weber R, Yerly S.

FUNDING:

Swiss National Science Foundation (grant # 33CS30_134277).

***University Medical Center Utrecht Dutch controls cohort***

DESCRIPTION:

These healthy Dutch individuals recruited as controls at the University Medical Center Utrecht for previous genetic studies (see Nat Genet. 2009;41(10):1083-7).

MEMBERS:

Jan H. Veldink, Leonard H. Van den Berg

FUNDING:

This project was supported by the Prinses Beatrix Fonds, VSB fonds, H. Kersten and M. Kersten (Kersten Foundation), The Netherlands ALS Foundation, and J.R. van Dijk and the Adessium Foundation. The research leading to these results has received funding from the European Community’s Health Seventh Framework Programme (FP7/2007-2013) under grant agreement 259867.

***The US military HIV Natural History Study (NHS)***

DESCRIPTION:

The US military HIV Natural History Study is a longitudinal epidemiological, observational, open cohort study collecting retrospective and prospective data in the U.S. Military active duty and Department of Defense (DoD) health care beneficiary HIV infected population.

MEMBERS:

Brian Agan, Mary Bavaro, Helen Chun, Nancy Crum-Cianflone, Cathy Decker, Connor Eggleston, Tomas Ferguson, Susan Fraser, Anuradha Ganesan, Joshua Hartzell, Joshua Hawley, Gunther Hsue, Arthur Johnson, Mark Kortepeter, Tahaniyat Lalani, Grace Macalino, Scott Merritt, Robert O’Connell, Jason Okulicz, Sheila Peel, Michael Polis, John Powers, Rose Ressner, Edmund Tramont, Tyler Warkentien, Amy Weintrob, Timothy Whitman, Michael Zapor

FUNDING:

Support for this work was provided by the Infectious Disease Clinical Research Program (IDCRP), a Department of Defense (DoD) program executed through the Uniformed Services University of the Health Sciences. This project has been funded in whole, or in part, with federal funds from the National Institute of Allergy and Infectious Diseases, National Institutes of Health (NIH), under Inter-Agency Agreement Y1-AI-5072. The content of this publication is the sole responsibility of the authors and does not necessarily reflect the views or policies of the NIH or the Department of Health and Human Services, the DoD or the Departments of the Army, Navy or Air Force.  Mention of trade names, commercial products, or organizations does not imply endorsement by the U.S. Government.

***The Wellcome Trust Case Control Consortium (WTCCC3) study of the genetics of host control of HIV-1 infection in the Gambia***

DESCRIPTION:

The WTCCC3 HIV-1 study involves scientists from The Gambia, Uganda and South Africa, working together with the genetics and analysis teams at the Wellcome Trust Centre for Human Genetics at Oxford to link genomic variants to how quickly people infected with HIV-1 develop the immune suppressed state which defines AIDS.

MEMBERS:

Adrian Hill, Fredrik Vannberg, Sir Andrew McMichael, Pontiano Kaleebu, Assan Jaye

FUNDING:

Funding for this work is provided by the Wellcome Trust Case Control Consortium. FV acknowledges the Georgia Institute of Technology Startup and Seed Grant funding, Cancer Treatment Centers of America (CTCA)

***The Western Australian HIV cohort Study***

DESCRIPTION:

The Western Australian (WA) HIV Cohort Study is a population-based, observational cohort study of HIV-infected patients in Perth, Australia.

MEMBERS:

Simon Mallal, David Nolan, Mina John, Olga Martinez, Martyn French, Elizabeth Phillips, Lloyd D’orsogna, Peter Hollingsworth, Lisa Boogerd
